# The spread of *Echinococcus multilocularis* in the Balkans is facilitated by the golden jackal

**DOI:** 10.3389/fvets.2026.1812243

**Published:** 2026-04-30

**Authors:** Aleksandra Uzelac, Katarina Breka, Aleksandra Penezić, Ilija Pantelić, Milica Kuručki, Neda Bogdanović, Tijana Kukurić, Nikola Betić, Jelena Karanović, Ivana Klun, Duško Ćirović

**Affiliations:** 1 Institute for Medical Research, University of Belgrade, Belgrade, Serbia; 2 Faculty of Biology, University of Belgrade, Belgrade, Serbia; 3 Faculty of Agriculture, Department of Veterinary Medicine, University of Novi Sad, Novi Sad, Serbia; 4 Institute of Meat Hygiene and Technology, Belgrade, Serbia; 5 Institute of Molecular Genetics and Genetic Engineering, University of Belgrade, Belgrade, Serbia

**Keywords:** carnivore, *Echinococcus multilocularis*, tapeworm, transmission, wildlife

## Abstract

**Introduction:**

Human alveolar echinococcosis (AE), caused by the zoonotic tapeworm *Echinococcus multilocularis*, is an emerging disease in the Balkans. The presence of this tapeworm has been detected in several wildlife species, including the golden jackal, a confirmed definitive host. The golden jackal is distributed throughout the Balkans, with a large resident population in Serbia. The aim of this study was to identify *Echinococcus* spp. circulating in golden jackals, identify regions with high occurrence of *Echinococcus multilocularis* and gain insight into the geographical spread of this tapeworm.

**Methods:**

The intestinal mucosa and feces of legally hunted golden jackals were examined for the presence of *Echinococcus* spp. adults and eggs. The mucosa was examined by sedimentation in saline, while taeniid eggs were isolated by zinc chloride flotation and collected after sequential mesh filtration. DNA was extracted from the adults/eggs and screened for the presence of DNA of the *Echinococcus granulosus sensu lato* complex and *Echinococcus multilocularis* using specific PCR methods.

**Results:**

*Echinococcus multilocularis* was detected in seven (5.7%) golden jackals (*n* = 122), *Echinococcus canadensis* G6-8, 10 was detected in two, while *Echinococcus granulosus sensu stricto* G1/3 was detected in one. Two areas with high occurrence of jackals infected with *Echinococcus multilocularis* were identified, one in the south (city of Niš and surrounding area) and another in the west (area including Čačak and Mount Zlatibor) of the country.

**Discussion:**

This study shows that *Echinococcus multilocularis* is the most frequent of the *Echinococcus* spp. tapeworms in golden jackals and widely distributed over Serbian territory. Of greatest concern to public health is the finding that 3/7 animals infected with *Echinococcus multilocularis* were detected in hunting grounds near cities (Niš and Čačak). The results of this study indicate that systematic surveillance and monitoring of golden jackals for the presence of *Echinococcus multilocularis* should be introduced in Serbia.

## 1 Introduction

Alveolar echinococcosis (AE) is a foodborne zoonotic disease caused by the cestode *Echinococcus multilocularis*, commonly known as the fox tapeworm. Environmental transmission of tapeworm eggs, shed by definitive hosts (Canidae), is the major route of infection for humans and other animals (small mammals, ungulates) which act as dead-end (aberrant) and intermediate hosts, respectively (1). In humans, infection occurs after accidental ingestion of eggs via contaminated food, commonly vegetables and berries. The eggs develop into larvae which subsequently establish vesicle-like cysts by infiltrating host tissue and creating lesions. Commonly, a single organ, the liver, is affected, but cysts can be established in multiple organs (2). The disease manifests after a long incubation period of several years, during which the tapeworm larvae continuously proliferate, thereby increasing the volume of the cyst(s) and lesions and exacerbating pathology of the affected organ(s). Metastases and spread to other organs further complicate the disease and potential treatment (1). Treatment of human AE involves removal of all of the affected tissue (organ resection) if possible and a life-long administration of anti-cestode drugs. Without appropriate and/or timely medical intervention, AE is often fatal. In terms of risk to public health, *E. multilocularis* was ranked highest in Europe using multicriteria decision analyses, primarily due to a significant case burden in central Europe (3). Echinococcosis is a notifiable disease in most European countries, however, few practice active surveillance of animal reservoirs, usually including only livestock (a source of infection for definitive hosts) and red foxes (4). Aside from foxes, which are crucial for the transmission and maintenance of *E. multilocularis* in central and western Europe, the golden jackal (*Canis aureus*) is another confirmed definitive host for *Echinococcus* spp. tapeworms. Foxes and especially golden jackals feed opportunistically by utilizing various natural and anthropogenic sources, thus supporting population growth in human-dominated landscapes and frequent incursion into urban environments (5–7). Both species therefore can facilitate a spillover of parasites which primarily cycle sylvatically, such as *E. multilocularis*, into the domestic cycle, thereby increasing the risk to public health. Over the last decades, the resident golden jackal population in the Balkans and the Pannonian region has been steadily increasing and concomitantly, the species’ European range has expanded into the northern and western parts of the continent. The golden jackal is expected to continue expanding its European range, as future climate change projections indicate increasingly suitable environmental conditions for the species (8). The golden jackal’s presence has been reported in at least 33 European countries (9). The population growth and range expansion of the golden jackal may be the largest documented for a carnivore species thus far (10, 11). In the Balkans, golden jackals have demonstrated a remarkable ability to adapt to different land use patterns in human altered landscapes, finding suitable habitats within agricultural areas and farmland in proximity to human settlements (12, 13). According to long term studies on jackal diet in Serbia, domestic ungulates, mainly pigs (carrion), and small mammals consistently emerged as the most important food sources, both of which facilitate continuous exposure to *Echinococcus* tapeworms (5, 14, 15). At present, there is no active or passive systematic surveillance of golden jackals for the presence of *Echinococcus* spp. tapeworms in Serbia nor any other country with a significant presence of these mesocarnivores. Therefore, all of the available data on the tapeworm species circulating in jackals and prevalence of infection originates from a limited number of scientific studies. A recent study of the movement patterns of GPS-collared jackals in a peripheral suburb of the capital, Belgrade, showed that they mostly move during the night and that males can routinely traverse a median daily distance almost twice that of females, approximately 2.3 km (11). This finding highlights the jackals’ capacity to spread *Echinococcus* tapeworm eggs into and within areas around human settlements. Several studies from the Balkans and in the neighboring Hungary reported the presence of *E. multilocularis* infection in golden jackals. A remarkably high presence of infection, 21.1% (*n* = 194), was detected in animals from the Transdanubia in Hungary recently (16). A few years earlier, *E. multilocularis* was detected in 14.1% (*n* = 64) of jackals from the northern-most Serbian province of Vojvodina, bordering Hungary (17), which is nearly identical to the finding from a decade ago, of 14.3% (*n* = 28) (18). A study from Bosnia and Herzegovina reported the presence of *E. multilocularis* in 6.8% (*n* = 44) of jackals (19), while Croatia reported 3.4% (*n* = 29) (20). The first human cases in the Balkans appeared in the last two decades, most recently in Croatia and Serbia, since 2017 and 2022, respectively (21, 22). Although the number of confirmed cases in Serbia remains low (three cases to date), a recent increase in Hungary—from approximately one to five cases per year—warrants concern (23). Interestingly, South Transdanubia, with a significant number of infected golden jackals, has been identified as a possible hyperendemic focus based on the human AE burden (23). Scientific literature from the region provides substantial evidence for a prominent role of the jackal in the transmission and/or maintenance of *Echinococcus* spp. As previously published reports on the presence of *E. multiloculari*s are available only from the Vojvodina province of Serbia, the aim of this study was to detect and identify *Echinococcus* spp. tapeworms in golden jackals throughout the entire territory of Serbia. The objective of this study was to gain insight into the geographical spread of *E. multilocularis*, identify regions which warrant introduction of jackal surveillance and monitoring for *E. multilocularis*, as well as highlight the role of the golden jackal in facilitating transmission of *Echinococcus* spp. tapeworms.

## 2 Materials and methods

### 2.1 Study design and sample collection

In Serbia, the golden jackal is classified as a game species with an open hunting season and may be hunted year-round (24). Ethical approval was not required for this study, as samples were obtained in accordance with the local hunting legislation. For this study, a collaboration agreement with the Hunters Association of Serbia (Lovački Savez Srbije - LSS) was signed in order to facilitate cooperation with local hunting organizations for the purposes of sampling. The request for cooperation was distributed by the LSS and local organizations which manage 25 public hunting grounds in different parts of the country agreed to provide samples on a voluntary basis. Hunting was done by licensed hunters. The number of carcasses collected from individual hunting grounds was based on the frequency and intensity of hunting, as well as availability of fresh carcasses (collected immediately after the hunt). All samples (*n* = 122) originated from animals hunted between October 2023 and February 2025. The GPS location of each dead animal was recorded, along with standard morphometric measures and sex for future studies. The carcasses were preferentially frozen whole at −20 °C. The carcasses remained frozen for a minimum of two weeks at −20 °C on average prior to the removal of the intestines. In rare circumstances, the intestines had to be removed in the field, packaged in plastic bags and leak-proof boxes and transported in a portable freezer to the laboratory immediately. All intestines remained frozen at −20 °C for several weeks at the laboratory to allow for batch processing.

### 2.2 Sample processing

The samples were allowed to semi-defrost at 4 °C overnight. The intestines were then cut into sections of at least 20 cm and opened longitudinally using surgical instruments. Any content was removed and stored in 50 mL conical tubes prior to scraping the mucosal lining using a 150 mm chisel shaped carbon PEEK probe (Ideal-tek S.A., Balerna, Switzerland). The mucosal scrapings and feces were collected in separate 50 mL centrifuge tubes.

### 2.3 Examination of the mucosa

Copious amounts of phosphate buffered saline (PBS) were added to the scraped mucosa, mixed well and then poured into sedimentation cups. The mixture was allowed to sediment 5–10 min prior to pouring off the supernatant. The sedimentation was repeated using fresh PBS several times until the sediment became clear, then transferred carefully into petri dishes and examined under an inverted microscope. Tapeworms which displayed gross morphological features characteristic of adult *Echinococcus* spp. were carefully collected and transferred into microcentrifuge tubes containing nuclease-free water. The worms were frozen at −20 °C until DNA extraction.

### 2.4 Zinc chloride flotation and sequential mesh filtration for taeniid egg retrieval

Up to 10 g of jackal feces were used for ZnCl_2_ flotation as described before (25, 26). The feces were first dissolved in 15 mL of dPBS-Tween20 solution (3%) and filtered through a tea strainer to remove large particles (bone fragments and bristles). The filtrate was centrifuged at 1600 g for 10 min, the supernatant was decanted and 15 mL of ZnCl_2_ solution with specific gravity of 1.45 g/cm^3^ was added before centrifuging for 30 min at 400 g. The supernatant was filtered sequentially through mesh filter cloth with pore size 50 μm and 22 μm (Franz Eckert GMBH, Waldkirch, Germany). The taeniid eggs captured on the 22 μm mesh filter were rinsed off with type 3 water using a squirt bottle and captured in clean 50 mL centrifuge tubes. The eggs were pelleted by centrifugation at 1600 g for 10 min, the supernatant was removed and the pellet was re-suspended in approximately 1 mL of nuclease-free water.

### 2.5 PCR screening for *Echinococcus* spp. DNA

Total nucleic acids were extracted from 30 μL of the egg pellet using 500 μL Trizol reagent, according to the manufacturer’s instructions (Ambion, Invitrogen, Waltham, MA, USA). Тhe samples were screened for the presence of *E. granulosus sensu lato* complex using real time qPCR (27), while *E. multilocularis* DNA was detected using a previously published conventional PCR protocol using primers EM-H15 and EM-H17 to amplify a 200 bp region of the 12S rRNA gene (28). Briefly, reactions were run in a total volume of 25 μL, which included 1X Multiplex Hot-Start PCR Master Mix (biotechrabbit, Berlin, Germany), 200 pmol each primer and 2 μL of the extracted DNA as a template. Positive (*E. multlocularis* DNA) and negative control (nuclease-free water) were included in each run. The reactions were visualized on agarose gels with EtBr. As a routine quality control measure, select products were sequenced to confirm the specificity of the EM-H15 and EM-H17 primers and validate the method internally.

### 2.6 Mapping and statistical analyses

The locations of jackal carcasses were documented and mapped using geographical information system (GIS) software QGIS 3.40.2. Confidence intervals (95% CI) were calculated using Wilson score intervals with continuity correction.

## 3 Results

**Figure 1:**
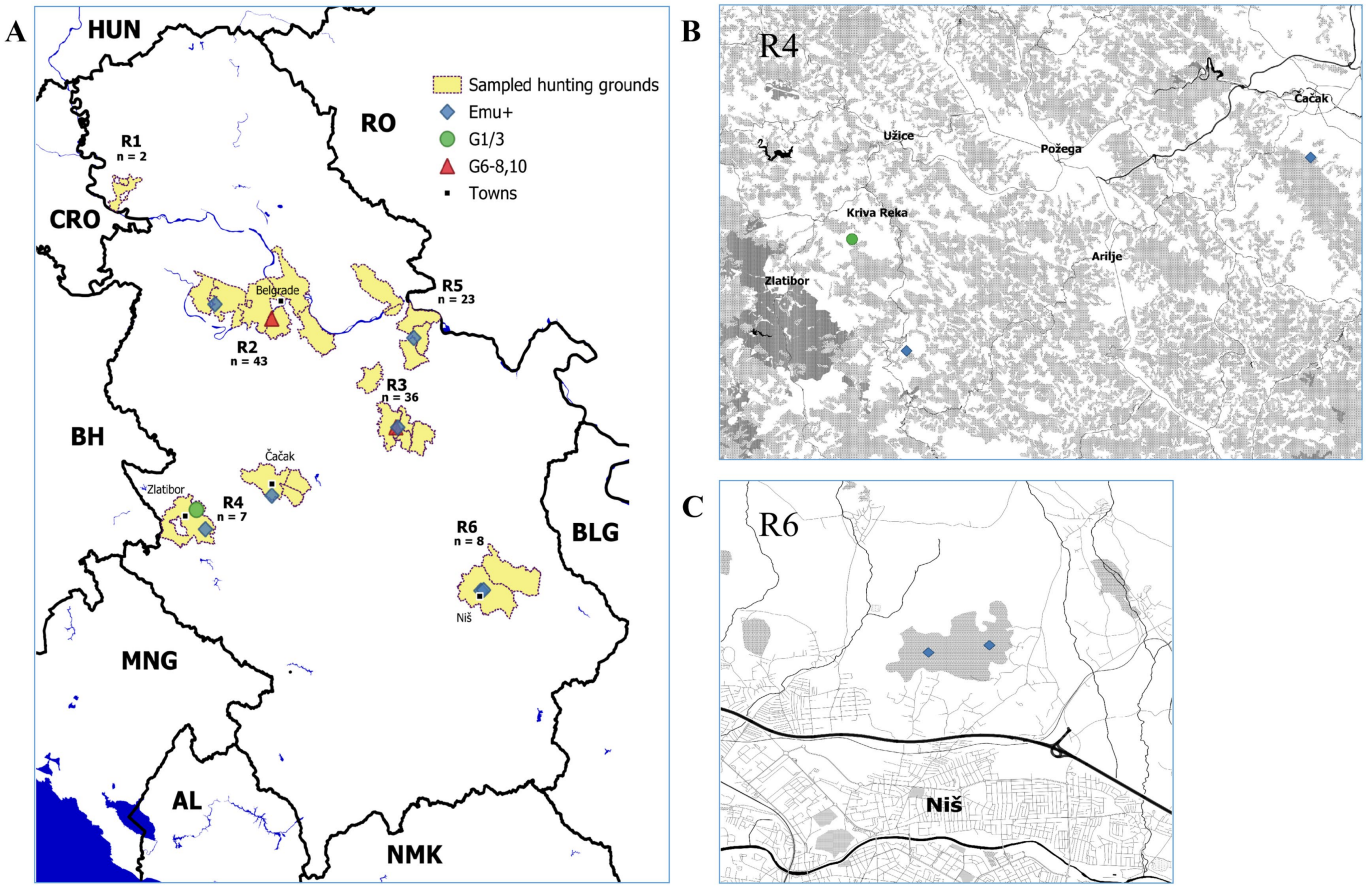
Sampled hunting grounds and distribution in R1-R6 with positive results of *Echinococcus* spp. detection. **(A)** Overall results showing the hunting grounds (shaded pale yellow) in R1-R6. The number of samples per R is indicated. Major settlements are indicated and shown as black squares. *Echinococcus multilocularis* positive samples are indicated by blue diamonds, *Echinococcus granulosus s. s.* (G1/3) by green circles, *Echinococcus canadensis* G6-8,10 by red triangles. **(B)** Close up of R4 around Mt. Zlatibor showing *Echinococcus multilocularis* occurrence near settlements. **(C)** Close up of R6 showing part of the city of Niš and *Echinococcus multilocularis* occurrence.

For this study, 122 golden jackal intestines were examined for the presence of *Echinococcus* spp. worms and eggs. The samples originated from 25 hunting grounds which form several clusters in different parts of the country (Figure 1A). The year, settlement closest to the GPS locations of the individual carcasses, hunting grounds from which each sample originated and the result of detection for each sample are shown in Supplementary Table 1. The numbers of samples obtained from individual hunting grounds were highly variable. The samples were grouped, based on the proximity of the hunting grounds, into several regions (designated as R1-R6) (Table 1). The purpose of the grouping was to provide mapping references for multiple, clustered hunting grounds from which the samples originated and to facilitate analysis, especially in the context of the distribution of *E. multilocularis*. The majority of samples, *n* = 43, originated from R2, represented by seven hunting grounds, followed by R3, *n* = 36, represented by five hunting grounds, and R5 with *n* = 23, also five hunting grounds. Very few samples were obtained from R1 (*n* = 2, represented by two hunting grounds), R6 (*n* = 8, represented by two hunting grounds) and R4 (*n* = 10, represented by four hunting grounds) (Table 1). The geographical distribution of R1-R6, the numbers of samples in each R as well as the detected *Echinococcus* spp. are indicated in Figure 1A. The human population size of settlements (cities, townships, villages) in R1-R6, which are situated within or are in the immediate vicinity of hunting grounds is indicated in Table 1. Adult worms were collected from two jackals, identified as *E. multilocularis* in both cases, while *Echinococcus* spp. eggs were present in ten golden jackals (8.2%; 95% CI: 4.2–14.9%) spread over R2-R6 (Figure 1A). Overall, *E. multilocularis* was detected in seven animals (5.7%; 95% CI: 2.5–11.9%), also scattered in R2-R6. *E. canadensis* (G6-8, 10) was detected in two animals from R2 and R3, while *E. granulosus sensu stricto* (G1/3) was detected in one animal from R4. Coinfections with different *Echinococcus* spp. were not detected. Despite few samples, in R4 and R6 relatively high proportions of animals infected with *E. multilocularis* were detected, 2/10 (95% CI = 3.5–55.8%) and 2/8 (95% CI = 4.4–64.4%), respectively (Figures 1B,C). Settlements in R4 and R6 closest to the infected animals, and approximate number of the resident human population are shown in Table 2.

**Table 1:** Overview and description of sampling regions, resident human population and results.

Sampling region	Description	Human population (52)	*N* (samples)	*N* positive (species)
R1	Two hunting grounds including the township of Bač and surroundings	11,317	2	none
R2	Seven hunting grounds, including city of Belgrade and surroundings	1,683,692	43	1 (*Echinococcus multilocularis*), 1 (*Echinococcus canadensis* G6-8, 10)
R3	Five hunting grounds including townships of Velika Plana, Svilajnac, Despotovac and surroundings	80,611	36	1 (*Echinococcus multilocularis*), 1 (*Echinococcus canadensis* G6-8, 10)
R4	Three hunting grounds including city of Čačak and surroundings	105,612	3	1 (*Echinococcus multilocularis*)
	One hunting ground including Mount Zlatibor resort area and surroundings	8,345	7	1 (*Echinococcus multilocularis*), 1 (*Echinococcus granulosus* G1/3)
R5	Five hunting grounds including township of Veliko Gradište and surroundings	18,988	23	1 (*Echinococcus multilocularis*)
R6	Two hunting grounds including city of Niš and surroundings	249,501	8	2 (*Echinococcus multilocularis*)
Total			122	7 (*Echinococcus multilocularis*), 2 (*Echinococcus canadensis* G6-8, 10), 1 (*Echinococcus granulosus* G1/3)

**Table 2:** Population at potential risk in areas with high *Echinococcus multilocularis* occurrence.

Sampling region	Closest settlement	Human population closest to locality of infected animals (52)	Proportion (positive animals/Total analyzed)
R4	Atenica (Čačak)	522	1/1
	Kriva reka	51	1/4
R6	Vinik (Niš)	1,429	2/7

## 4 Discussion

This study, based on carcasses collected from 25 hunting grounds in different parts of Serbia, showed that 8.2% of the examined golden jackals (10/122) harbored *Echinococcus* spp. eggs, while adult *E. multilocularis* worms were recovered from the mucosa of two animals. The eggs collected from the animals from which the adults were recovered were also identified as *E. multilocularis*. The presence of *E. multilocularis* was absolutely dominant (7/10), detected in 5.7% of the total number of golden jackals examined, while *E. canadensis* G6-8,10 was detected in two and *E. granulosus s. s*. G1/3 in just one animal. The results presented here indicate that the occurrence of *E. multilocularis* is more frequent than *E. granulosus s. l.* complex in golden jackals.

Serbia’s territory is dominated by human-altered landscapes, characterized by modifications of natural ecosystems of varying degree of intensity, which result in structural, compositional and ecological changes of different magnitude. The landscapes surrounding the hunting grounds R1-R6 are mosaics of arable land, forest/grassland, human settlements (ranging from cities to villages), industrial facilities and various infrastructure (buildings, roads, irrigation canals, drainage systems). Hunting grounds thus provide habitats and feeding grounds for wildlife in close proximity to human settlements and other areas characterized by human presence. The occurrence of *Echinococcus* spp. in golden jackals represents a potential risk for human infection and as infected animals may shed eggs over a period of time, findings of even few infected animals, should be acknowledged in the context of public health, especially due to the severity of AE.

The variability in occurrence of *Echinococcus* spp. from R1-R6 could perhaps be attributed to varying degrees of exposure of golden jackals to sources of infection, such as infected wildlife and/or livestock. Findings of hydatid cysts in livestock species from Serbia were previously reported at the slaughter line, yet unfortunately, the species of the infecting tapeworm was usually not identified by specific genetic sequences (PCR), but rather the reports are based predominantly on simple presence of cysts and rarely, gross pathology and/or cyst morphology (29, 30). Based on a scientific study from a decade ago, which did use molecular techniques, species of the *E. granulosus s. l.* complex were detected in domestic pigs, cattle and sheep from slaughterhouses, specifically *E. granulosus* genotypes G1, 2, 3 and *E. canadensis* genotype G7. Many of the infected animals originated from western Serbia (Mount Zlatibor region) and the vicinity of the city of Niš (31), localities within the R4 and R6 designated regions, respectively. Despite the fact that a specific molecular assay for *E. multilocularis* was not done in the aforementioned study, all of the cyst material analyzed yielded results, thus suggesting that *E. multilocularis* was not present among those samples. More recently, however, *E. multilocularis* was detected in a domestic pig liver from a slaughterhouse in central Serbia (32), indicating that this tapeworm species circulates domestically, albeit at a low frequency. The first record of disease caused by *E. multilocularis* in wildlife in Serbia was reported over a decade ago in a beaver (33), followed by detection of *E. canadensis* G7 in two wild boar (34). The presence of *E. multilocularis* in other species of wildlife suitable as prey for golden jackals, such as small mammals, has not been extensively investigated.

In terms of transmission of *Echinococcus* spp. to humans, historically, dogs (owned and stray) have been implicated as important for *E. granulosus s. s.,* as reported prevalences in dogs ranged from roughly 13% all the way up to 70%. However, confirmation by molecular methods for the presence of *Echinococcus* spp. DNA as well as identification of the tapeworm species was not done (35–40). A recent study of 382 owned dogs (hunting, guard, pet) identified taeniid eggs by morphology after conventional flotation using NaCl and/or ZnSO_4_ in merely 1.3% (five dogs) which were, as a common precautionary practice, attributed to *E. granulosus* (41). In light of that, it is likely that the aforementioned findings also reflect the presence of taeniid eggs. Thus, the occurrence of taeniid eggs in dogs appears to have dropped significantly over the last decades. Prior to 2023, human echinococcosis cases in Serbia were reported only as CE, mostly attributed to *E. granulosus s. s*. and a single case (confirmed by molecular methods) to *E. canadensis* G7 (42–44). The number of cases reported has varied annually, displaying a generally decreasing trend, with occasional annual spikes (45, 46). Collectively, these findings make it difficult to ascertain the significance of dogs in the transmission of *Echinococcus* spp. tapeworms to humans with any certainty.

Despite the fact that samples of foxes and dogs were not examined for this study, the presence of *E. multilocularis* in golden jackals, indicates that this tapeworm species is in circulation near human settlements, which provides an opportunity for human infection. According to a study by Moloi et al. (47), the proportion of fertile worms and mean egg production in worms from foxes and golden jackals were similar, despite lower numbers of mature eggs in worms from golden jackals. The findings suggest that while foxes appear to be optimal hosts for *E. multilocularis*, the golden jackals’ contribution to the maintenance and transmission of this tapeworm cannot be ignored. Given the overlap in trophic niche as well as habitat preferences of these two mesocarnivores (48), the golden jackal may in fact distribute and spread the eggs in the environment similar to foxes.

To date, only the district of Srem has been identified as a hotspot for *E. multilocularis*, due to a high occurrence in foxes (13%) and golden jackals (14.3%) and the fact that human AE cases in Serbia were thus far diagnosed only in patients residing in that district in the province of Vojvodina (22, 49, 50). The findings presented here in golden jackals from the locality of Vinik, close to the city of Niš (R6), are particularly alarming in the context of public health, as the proportion of animals harboring *E. multilocularis* eggs was highest, albeit a low number of samples. Furthermore, 7/8 examined jackals from R6 were shot in close proximity to urban infrastructure and residential areas (Figure 1C). Additional samples of jackals from a wider area of the suburban zone of Niš need to be examined to better ascertain the risk of infection for the city population. Similarly, R4, which includes a much larger territory in western Serbia (Figures 1B,C) showed a higher occurrence of *Echinococcus* spp. in examined animals. Greater sampling efforts, primarily targeting animals from hunting grounds near townships and villages in the foothills of Mount Zlatibor, need to be undertaken in the future to estimate risk to the human population. Targeted approaches to assess the risk to the human population particularly in R4 and R6, which include detection of *E. multilocularis* eggs in the environment (soil and water) as well as locally grown vegetables and fruits, will be considered in the future.

A limitation of the study was the geographical coverage of the sampled areas, most likely due to differences in hunting intensity. Hunting intensity is based on the population size and density of golden jackals in different parts of the country, but as they are not a game species and have no value as trophies, they may also be of generally low priority to hunters. While analyis of hunting bag data would allow evaluation of hunting intensity, a systematized, periodical collection is not mandated and a centralized database is not available. Additionally, there are no reliable estimates of the population size and density on a regional or national level. The results of this study have to therefore be interpreted with caution and should not be taken as a reflection of the epidemiological situation in the country. An inherent constraint in legal sampling of wildlife is the spatial restriction in terms of hunting grounds, which have been grouped into R1-R6 here. Studies of the golden jackals’ movement ecology suggest that individuals may disperse over considerable distances, thus excursions into areas outside of hunting grounds cannot be ruled out (51). The results may thus not reflect the true spread of *E. multilocularis* and risk for human infection. Despite the limitations and constraints, this study nonetheless provides an insight into the distribution of *E. multilocularis* in different parts of the country and highlights the significance of golden jackals as definitive hosts of this tapeworm species.

Collectively, the findings indicate that in regions with large population size and/or high density of golden jackals, such as the Balkans, it is conceivable that the golden jackal may replace the fox as the major definitive host of *E. multilocularis.* In light of that, the implications for public health in Serbia are potentially serious and countrywide systematic surveillance and monitoring of *E. multilocularis* in golden jackals is urgently needed.

## Data Availability

The original contributions presented in the study are included in the article/Supplementary material, further inquiries can be directed to the corresponding author/s.
